# Clinically Combating Reward Deficiency Syndrome (RDS) with Dopamine Agonist Therapy as a Paradigm Shift: Dopamine for Dinner?

**DOI:** 10.1007/s12035-015-9110-9

**Published:** 2015-03-10

**Authors:** Kenneth Blum, Marcelo Febo, Panayotis K. Thanos, David Baron, James Fratantonio, Mark Gold

**Affiliations:** 1 Department of Psychiatry and McKnight Brain Institute, College of Medicine, University of Florida, P. O. Box 100256, Gainesville, FL 32610-0256 USA; 2 Human Integrated Services Unit, Center for Clinical and Translational Science, Department of Psychiatry, College of Medicine, University of Vermont, Burlington, VT USA; 3 Division of Applied Clinical Research, Dominion Diagnostics, LLC, North Kingstown, RI USA; 4 Department of Addiction Research and Therapy, Malibu Beach Recovery Center, Malibu, CA USA; 5 Behavior Neuropharmacology and Neuroimaging Laboratory, Department of Psychology, SUNY at Stony Brook, Stony Brook, NY USA; 6 Department of Psychiatry, Keck School of Medicine, University of Southern California, Los Angeles, CA USA; 7 Keck School of Medicine, University of Southern California, Los Angeles, CA USA; 8 Department of Research, Rivermernd Health, Atlanta, GA USA

**Keywords:** Dopamine, Reward deficiency syndrome (RDS), Agonistic therapy, Dopamine societies, Genetics and epigenetics

## Abstract

Everyday, there are several millions of people that are increasingly unable to combat their frustrating and even fatal romance with getting high and/or experiencing “normal” feelings of well-being. In the USA, the FDA has approved pharmaceuticals for drug and alcohol abuse: tobacco and nicotine replacement therapy. The National Institute on Drug Abuse (NIDA) and the National Institute on Alcohol Abuse and Alcoholism (NIAAA) remarkably continue to provide an increasing understanding of the intricate functions of brain reward circuitry through sophisticated neuroimaging and molecular genetic applied technology. Similar work is intensely investigated on a worldwide basis with enhanced clarity and increased interaction between not only individual scientists but across many disciplines. However, while it is universally agreed that dopamine is a major neurotransmitter in terms of reward dependence, there remains controversy regarding how to modulate its role clinically to treat and prevent relapse for both substance and non-substance-related addictive behaviors. While the existing FDA-approved medications promote blocking dopamine, we argue that a more prudent paradigm shift should be biphasic—short-term blockade and long-term upregulation, enhancing functional connectivity of brain reward circuits.

*Mille viae ducunt homines per saecula Romam* (A thousand roads lead men forever to Rome) in *Liber Parabolarum,* 591 (1175), by Alain de Lille.

Scientific explorations from around the globe agree that substance and non-substance-seeking behaviors are considered an endemic societal problem affecting multimillions. Certainly, we have come a long way since Bill Wilson and Dr. Bob Smith began their crusade in 1933 embracing the “Cambridge” theories and doctrines, resulting in the most powerful anti-alcohol/drug program and fellowship in the world—Alcoholics/Narcotic Anonymous. Many years after Jelnick’s famous article in 1956 providing the medical profession with the “concept of alcoholism as a disease,” the American Society of Addiction Medicine (ASAM) espoused a new definition of addiction indicating that “Addiction is a primary, chronic disease involving brain reward, motivation, memory and related circuitry”; it can lead to relapse, progressive development, and the potential for fatality if not treated. While pathological use of alcohol and, more recently, psychoactive substances has been accepted as an addictive disease, developing brain science has set the stage for inclusion of the process addictions, including food, sex, shopping, and gambling problems, in a broader definition of addiction as set forth by the American Society of Addiction Medicine in 2011 [1].

To carry out this review, we searched a number of important databases including the following: filtered resources—Cochrane Systematic Reviews, DARE, PubMed Central Clinical Queries, National Guideline Clearinghouse; unfiltered resources—PsychINFO, ACP PIER, PsychSage, PubMed/MEDLINE. The major search terms included the following: dopamine agonist therapy for addiction, dopamine agonist therapy for reward dependence, dopamine antagonistic therapy for addiction, dopamine antagonistic therapy for reward dependence. Our results produced the following: *dopamine agonistic therapy for addiction*—Cochrane Systematic Reviews 0, DARE 0, PubMed Central Clinical Queries 9, National Guideline Clearinghouse 0, PsychINFO 0, ACP PIER 83, PsychSage 15, PubMed/MEDLINE 501; *dopamine agonist for addiction*—Cochrane Systematic Reviews 3, DARE 3, PubMed Central Clinical Queries 10, National Guideline Clearinghouse 0, ACP PIER 0, PsychSage 15, PubMed/MEDLINE 13; *dopamine agonistic therapy for reward dependence*—Cochrane Systematic Reviews 0, DARE 0, PubMed Central Clinical Queries 1, National Guideline Clearinghouse 0, PsychINFO 0, ACP PIER 0, PsychSage 0, PubMed/MEDLINE 62; *dopamine agonist for reward dependence*—Cochrane Systematic Reviews 0, DARE 0, PubMed Central Clinical Queries 337, National Guideline Clearinghouse 0, PsychINFO 1, ACP PIER 0, PsychSage 0, PubMed/MEDLINE 120 (see Fig. 1); *dopamine antagonistic therapy for addiction*—Cochrane Systematic Reviews 0, DARE 0, PubMed Central Clinical Queries 0, National Guideline Clearinghouse 0, PsychINFO 0, ACP PIER 0, PsychSage 0, PubMed/MEDLINE 633. Clearly, we utilized a combination of PubMed Central Clinical Queries and PubMed/MEDLINE for our reliable review search as well as author searches based on personal knowledge of the field.

**Fig. 1:**
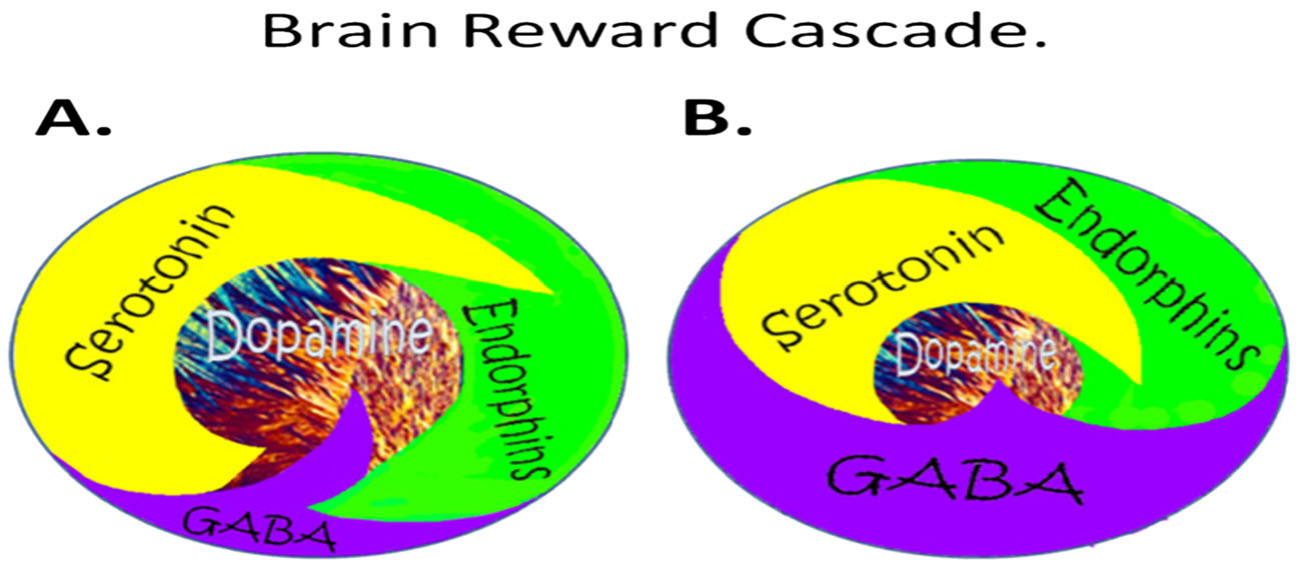
Schematic of the brain reward cascade: normal and abnormal representation. **a** The normal physiologic state of the neurotransmitter interaction at the mesolimbic region of the brain. Briefly, serotonin in the hypothalamus stimulates neuronal projections of methionine enkephalin in the hypothalamus that, in turn, inhibits the release of GABA in the substantia nigra, thereby allowing for the normal amount of dopamine to be released at the nucleus accumbens (NAc) reward site of the brain. **b** Hypodopaminergic function of the mesolimbic region of the brain. The hypodopaminergic state is due to gene polymorphisms as well as environmental elements (epigenetics), including both stress and neurotoxicity from aberrant abuse of psychoactive drugs (i.e., alcohol, heroin, cocaine, etc.) and genetic variables [2]

According to Belcher et al., drug addiction is characterized by a compulsive drive to take drugs despite serious negative consequences and is a disorder that involves complex interactions between genetic and environmental variables [3]. For example, undoubtedly, heroin addiction is a complex phenomenon of the brain involving both affective and cognitive processes [4]. It has been found that in heroin-dependent individuals, there is increased white matter intensity in the frontal area and decreased gray matter density in the bilateral prefrontal cortices and in the temporal regions compared to healthy subjects [5]. It was also found that there is a high accuracy in the activation pattern differences between heroin-dependent subjects and healthy individuals during resting-state brain activities. These differences of activation patterns included orbitofrontal cortex (OFC), hippocampal/parahippocampal region, amygdala, caudate, putamen, as well as the insula and thalamus [6].

Moreover, similar effects were found for alcohol as well. Specifically, Luhar et al. [7] compared non-smoking non-alcoholics—alcoholics who smoke had volumetric abnormalities in pre- and paracentral frontal cortical areas and rostral middle frontal white matter, parahippocampal and temporal pole regions, the amygdala, the pallidum, the ventral diencephalic region, and the lateral ventricle. The comorbid group performed worse than non-smoking non-alcoholics on tests of executive functioning and on visually based memory tests. Similar findings have been observed with chronic cocaine [8], food abuse [9], and other addictive behaviors [10].

Importantly, Volkow et al. [11] proposed a map consisting of four circuits involved in drug abuse and possibly reward behaviors (i.e., addiction): (1) reward, located in the nucleus accumbens (NAc) and ventral pallidum; (2) motivation/drive, located in the OFC and subcallosal cortex; (3) memory and learning, located in the amygdala and hippocampus; and (4) control, located in the prefrontal cortex and anterior cingulate gyrus (ACC).

Our current knowledge indicates that whereas aberrant craving behavior resides in the caudate-accumbens brain region, loss of control and thus relapse occur in the cingulated gyrus [12]. Moreover, Thanos et al. [13] as well as Rothman et al. [14] independently suggested that dopamine agonist therapy by either increasing D2R availability or enhanced dopamine release could be a useful therapeutic adjunct for the treatment of cocaine, alcohol, heroin addictions, as well as for obesity, attention deficit disorder, and depression or reward deficiency syndrome (RDS) behaviors [15].

We hypothesized that the putative natural anti-craving/anti-relapse compound KB220Z™ as reviewed by Chen et al. [16] may activate dopaminergic pathways. This complex is a neuroadaptagen comprising amino acid neurotransmitter precursors and enkephalinase-catecholamine-methyl-transferase (COMT)-MAO-inhibition therapy called neuroadaptagen amino acid therapy (NAAT). A number of KB220 variants, developed in the late 1980s, have been the subject of approximately 27 clinical trials (see review [17]).

Ongoing research on KB220Z™ repeatedly confirms the numerous clinical effects that ultimately result in significant benefits for victims having genetic antecedents for all addictive, compulsive, and impulsive behaviors [18]. In earlier research in the USA using qEGG in protracted abstinence in psychostimulant-dependent subjects, this compound showed an increase in both alpha and low beta bands in the OFC and cingulated gyrus, overcoming qEEG abnormalities [19] also seen with alcoholics and heroin addicts [20].

We are cognizant that risk for relapse has now been linked to hypodopaminergic genetics. Dani et al. [21] suggested that withdrawal from nicotine induces a hypodopaminergic state, but there is a relative increase in the sensitivity to phasic dopamine release that is caused by nicotine. Therefore, acute nicotine reexposure causes a phasic dopamine (DA) response that more potently reinforces relapse to smoking during the withdrawal period. The supersensitivity to DA in terms of relapse has been the subject of a number of articles, whereas our laboratory suggested that relapse to psychoactive drugs is due to a process called *deprivation-amplification relapse therapy (DART)* [22]. We proposed that low D2 receptor density and polymorphisms of the D2 gene are associated with risk for relapse of substance abuse, including alcohol dependence, heroin craving, cocaine dependence, methamphetamine abuse, nicotine sensitization, and glucose craving. We further proposed that a putative physiological mechanism that may help to explain the enhanced sensitivity following intense acute dopaminergic D2 receptor activation is *denervation supersensitivity*. This concept is in agreement with the work of Dahlgren et al. [23] who reported that alcoholic carriers of the *Taq A1* allele of the dopamine D2 receptor gene compared to *Taq A2* allele carriers significantly relapse at a higher rate, suggesting a hypodopaminergic trait. Unfortunately, *Taq A1* allele alcoholics have a significant enhanced mortality rate [24] as well as cocaine addicts [25]. Historically, the notion that low dopaminergic function is linked to substance and non-substance-seeking behavior is well known, and this concept dates back to the *dopamine depletion hypothesis* espoused by Dackis and Gold [26] to explain cocaine relapse. More recently, others have indicated that carriers of the dopamine D2 receptor *Taq A2* allele have a reduced risk for all RDS behaviors including glucose craving and as such is protective [27, 28].

Over the last decade, a number of scientists pioneered the controversial concept that food addiction and drug addiction constitute common neurobiological mechanisms [29, 30]. Avena [31] reported on similar neurochemical changes during withdrawal from both heroin and sugar binging. The role of dopamine release and sugar binging seems interconnected as evidenced by the attenuation of palatable food seeking by an inhibitor of aldehyde dehydrogenase inhibitor-2 which also blocks DA release at the NAc [32]. While this may have some clinical relevance in the short term (i.e., reducing sugar binging), we caution long-term benefits because of anti-reward/dark side of addiction/deficiency of dopaminergic function [33, 34] and potential mood changes including suicide ideation [35]. Alternatively, we propose herein that dopamine agonistic, not antagonistic, therapy in the long term will be more prudent. We must ask the following question: Are we serving enough “dopamine for dinner”?

In earlier days, it was true that all roads did lead to Rome. This simple truth is not too dissimilar when we consider the *Homo sapiens* reward circuitry of the brain. Based on numerous experiments in the scientific literature, well over 20,000 entries, the major reward neurotransmitter pathway (road) indeed is DA [36].

The role of dopamine and mind function has been fraught with controversy but arguably very interesting and mind expanding. There are many unanswered questions related to what makes us human and what drives our unique behaviors. While many brain theories have focused on the role of brain size and genetic adaptations, Fred Previc, a scientist living in San Antonio, TX, explored the provocative and very speculative concept of a “dopaminergic society” [37].

According to Previc, the *dopaminergic mind hypothesis* seeks to explain the differences between modern humans and their hominid relatives by focusing on changes in dopamine. It theorizes that increased levels of dopamine were part of a general physiological adaptation due to an increased consumption of meat around two million years ago in *Homo habilis* and later enhanced by changes in diet and other environmental and social factors beginning approximately 80,000 years ago. Under this theory, the “high-dopamine” personality is characterized by high intelligence, a sense of personal destiny, a religious/cosmic preoccupation, an obsession with achieving goals and conquests, an emotional detachment that in many cases leads to ruthlessness, and a risk-taking mentality. High levels of dopamine are proposed to underlie increased psychological disorders in industrialized societies. According to this hypothesis, a dopaminergic society is an extremely goal-oriented, fast-paced, and even manic society, “given that dopamine is known to increase activity levels, speed up our internal clocks, and create a preference for novel over unchanging environments.” In the same way that high-dopamine individuals lack empathy and exhibit a more masculine behavioral style, dopaminergic societies are “typified by more conquest, competition, and aggression than nurturance and communality.”

In our view, the lack of brain dopamine function (dopamine deficiency) either due to genetics and/or environmental elements (epigenetics) could lead to a “hypodopaminergic society” whereby the individual may have a reduced cognition and memory, poor executive function, lack of motivation or achieving goals, low spirituality, liberal non-partisan views, selfishness, high novelty seeking, substance and behavioral reward seeking, lack of attention span, violent behavior, compromised well-being and blunted ability to achieve pleasure states, erotic love styles (less romantic in nature), enhanced immature defense style (lying), lowered pain tolerance, higher percent body fat, less energy, and basically unhappy (see brain reward cascade schematic Fig. 1).

Although behavioral evidence and some indirect anatomical evidence revealed by the work of SI Rapoport from the Laboratory of Neurosciences, National Institute on Aging, National Institutes of Health, Bethesda, MD (e.g., enlargement of the dopamine-rich striatum in humans), support a dopaminergic expansion in humans [38], according to MA Raghanti and associates from the Department of Anthropology at Kent State in Ohio, there is still no direct evidence that dopamine levels are markedly higher in humans relative to other apes [39]. However, recent discoveries about the seaside settlements of early man may provide evidence of dietary changes consistent with this hypothesis [40].

The possibility does exist that prehistoric ancestral species over two million years ago carried the low dopamine brain function due to low dopamine receptors. It should be noted that dopamine functions as a neurotransmitter activating the five known types of dopamine receptors—D1–D5—and their variants. Dopamine from l-tyrosine found in meat is produced in several areas of the brain, including the “brain reward” (nucleus accumbens) site located in the reptilian old brain region called the mesolimbic system. Scientists now know that there are two major variant forms of the human gene, for example, the dopamine D2 receptor gene (DRD2) that regulates the synthesis of D2 receptors and the most widely studied gene that accounts for major aspects of modern human behavior [41]. Specifically, there is DRD2 A2 form which in today’s world is considered the “normal” variation carried by two thirds of the US population, whereas the DRD2 A1 form carried by one third of today’s US population results in 30–40 % lowered D2 receptors and as such leads to a subset of approximately 100 million people that constitute the “low-dopamine society” [42]. It is parsimonious to consider that the older gene form (DRD2 A1) leading to low dopamine function may have afforded certain survival benefits, but as *H. habilis* increased their meat consumption, feeding the brain with the needed l-tyrosine to synthesize more dopamine required to overcome the D2 receptor deficit, a new society was born—the “high-dopamine society” carrying the DRD2 A2 form of this gene. The differences between carriers of the low-dopamine-expressing gene form A1 compared to the normal or higher expression of the dopamine D2 receptor gene A2 have been shown to be protective against ADHD, a subtype of RDS behavior [28].

Understanding that low dopamine function leads to impulsive, compulsive, and addictive behaviors paves the way to defining addiction as a brain disorder involving impairments in the so-called reward circuitry. This new definition of addiction has been now adopted by the American Society of Addiction Medicine (ASAM) founded by the San Franciscan visionary David E. Smith [1]. This new definition is based in part on the initial conceptualization of one of us (KB), who in 1995 coined “reward deficiency syndrome” (RDS) [43], a term to define common genetic antecedents for a predisposition for aberrant substance and behavioral seeking. This list is remarkable, and it may lead to alcoholism, opiate dependence, psychostimulant abuse (e.g., cocaine), nicotine dependence, glucose binging and overeating, inability to focus (ADHD and other spectrum disorders), pathological gambling, excessive Internet gaming, sex addiction, and obsessive-compulsive disorder, among other repetitive known behaviors.

While having any brain genetic deficit in the reward site may predispose an individual to a higher risk for RDS, it is always the combination of our genes and the interaction with environmental elements that predict not only addictive behaviors in general but specificity of the type of drug or behavior of choice. Using a special mathematical formulation (Bayesian—developed by a sixteenth-century monk) to predict the value of carrying for example the DRD2 A1 (low D2 receptors) at birth and subsequent lifetime, the risk of any RDS behavior has been determined to be as high as 74 % [44].

Thus, our provocative hypodopaminergic RDS theory is highly impacted by what scientists like Steve Sussman of Southern California University call epigenetic factors (environmental) rather than just genetic factors and translation to preventive strategies [45] for school children and others [46] involved in epigenetics from a cellular exploration. So, the take-home message is that while one is not doomed because of their genes to become an addict, they are definitely at high risk and as such may require this genetic knowledge earlier than later in life.

The FDA-approved medications for alcohol, drugs, and food are at best only moderately effective and in some cases counter to promoting “dopamine homeostasis” including narcotic antagonists and acamprosate, a NMDA antagonist, for example, utilized to reduce drug-induced euphoria via attenuated neuronal dopamine release at the NAc [47, 48]. We argue the work of Shelton et al. [49] who suggested that the known anxiolytic buspirone may reduce the likelihood of relapse to cocaine and methamphetamine use under some conditions, especially for adolescents. Our concern with this concept is that understanding the pharmacological complexity of this substance involving serotonin 5-HT1A receptor partial agonist activity and dopamine D2, D3, and D4 receptor antagonist effects thereby reducing availability of dopamine seems counterintuitive.

Our laboratory extensively published on the concept of dopamine agonist therapy to treat iatrogenic opioids and even prevent RDS behaviors [50]. A major challenge in effectively utilizing chronic dopamine agonist therapy is the manner in which powerful D2 agonists like bromocriptine work to ultimately reduce D2 receptor density [51]. In agreement with dopamine agonist therapy is the recent work of Maguire et al. [52] showing the interactive role of D1 and D2 receptor activation in affecting synaptic GABAA receptors within the NAc altering GABA’s inhibition of medium spiny neurons subsequently influencing behavioral responses to cocaine. While utilization of dopamine agonists may be complicated, it is of interest that Czoty and Nader [53] clearly showed the differences between a low-dopamine agonist compared to a high-dopamine agonist in terms of blocking food-cocaine choice in socially housed male cynomolgus monkeys primarily favoring the low agonist compared to the high agonist.

Moreover, Radke and Gewirtz [54] showed that increasing dopamine receptor activity in the NAc shell utilizing known dopamine D2 agonists significantly ameliorates anxiety during withdrawal from morphine and nicotine. This work is underscored by an earlier work from Thanos et al. [55–57] showing attenuation of alcohol and cocaine intake by gene therapy. Along similar lines, Crunelle et al. [58] showed that varenicline (a partial agonist at α4β2 nicotinic acetylcholine receptors) significantly increases the availability of D2/D3 receptors and concomitantly reduces nicotine-seeking behavior. Interestingly, van Rijn et al. [59] found that the delta opioid receptor agonist (selective DOR agonists) can decrease anxiety-like behavior and reduce ethanol consumption. This is important when you factor in the interaction of opioid receptor activity and inhibition of GABA transmission at the substantia nigra [60]. In further support of our hypothesis, Carvalho et al. [61] observed that long-term haloperidol administration to mice resulted in enhanced addiction-related behaviors due to a suspected supersensitivity induced by prolonged D2 blockade favoring our DART concept [22].

It is also known that cocaine reinstatement occurs only with D1 receptor agonists, not D2 receptor agonists, and as such Graham et al. [62] provide clear evidence for this differential effect which is underscored by others [63]. This work is highlighted by Caine et al. [64] who reported that in wild-type mice, pretreatment with the D2-like antagonist eticlopride increased rates of self-administration of high doses of cocaine, and the D2-like agonist quinelorane served as a positive reinforcer when substituted for cocaine, supporting dopamine D2 agonist therapy.

In humans, Schmidt et al. [65] reported that the dopamine D2 agonist lisuride prolonged the latency of relapse, whereby the authors suggested that alcoholics may relapse due to decreased dopamine function. In addition, Koob’s group [66] showed that pretreatment with the dopamine D2 receptor agonist bromocriptine attenuated the cocaine-induced increase in responding for the cocaine-associated cue. In contrast, pretreatment with low doses of SDZ 208-911, a dopamine D2 partial agonist reducing D2 receptor availability, further potentiated the cocaine-induced response. Further work in humans by Lawford et al. [67] showed that D2 agonist therapy with bromocriptine prevented relapse in alcoholics especially those that carry the DRD2 A1 allele, indicating that low dopamine function responds best compared to normal D2 receptor density in A2 carriers.

The work of Ng and George [68] firmly supports a dopamine hypothesis for ethanol abuse in the genetically ethanol-preferring C57 mouse as argued by Blum et al. in earlier experiments [69]. While there is still controversy concerning how best to treat RDS as it relates to drug seeking and relapse especially in terms of dopaminergic function [70], based on our experience involving a natural putative dopamine agonistic pharmacological profile [17] and human neuroimaging studies (qEEG and fMRI) [17, 19, 20], we are hereby proposing that following required research, short-term blockade of D2 receptors is prudent but long-term treatment should activate, not block, D2 receptors.

Understanding the true role of dopamine in escalating, for example, cocaine has been the recent subject of investigation by a number of investigators. Weiss’s group provided strong evidence that attenuated dopamine actions in the core (but not the shell) of accumbens result in greater cocaine intake—shorter interresponse times—during periods of self-administration [71]. Conversely, they find that enhanced dopamine actions in the core (but not the shell) result in decreased intake—longer interresponse times [72]. In agreement with these findings, Willuhn et al. [73] surprisingly found that phasic dopamine decreased in the ventromedial striatum (VMS) as the rate of cocaine intake increased, with the decrement in dopamine in the VMS significantly correlated with the rate of escalation. Moreover, the administration of the dopamine precursor l-DOPA at a dose that replenished dopamine signaling in the VMS reversed escalation, thereby demonstrating a causal relationship between diminished dopamine transmission and excessive drug use. While this work is intriguing, it may not morph other theories such as the surfeit rather than deficit dopamine function; however, it does seem to support dopamine-based agonistic modalities as suggested by Willuhn et al. [73]. Along these lines, Caprioli et al. [74] correctly conclude that, at present, there are no FDA-approved medications for cocaine addiction. However, several clinical studies have suggested that agonist-based substitution treatment (for example, prescription oral amphetamine) decreases illegal cocaine use [75]. While we do not clinically agree with the amphetamine approach (chronically leading to downregulation of D2 receptors as seen with other D2 agonists [76]), it does point out the importance of dopamine agonist therapy compared to current theories utilizing dopamine antagonistic therapy to treat cocaine escalation during protracted abstinence.

Epigenetics, an emerging area in the neuroscience field, has provided important clues about how drugs of abuse including alcohol interact with epigenome and modulate the genetic functions and regulate addictive endophenotypes [77, 78]. Epigenetic modifications, such as histone and DNA chemical modifications, also play an important role in neurodevelopment [79, 80]. Epigenetics explains how environmental and psychological factors regulate the activity of our genome without inducing changes in the DNA sequence. It has been suggested that epigenetics mediates our behavior, in part, and has long-term effects on the regulation of the genome function. In fact, González-Pardo and Pérez Álvarez [81, 82] indicate that epigenetics impacts nature-nurture, genotype-phenotype, or pathogenesis-pathoplasty.

One important outcome of this concept has been adopted by a new cook book entitled *Dopamine for Dinner* [76] responding to the question: Are we serving enough dopamine for dinner? As scientists interested in our most troublesome issues in psychiatry, reward deficiency, and sports medicine, we encourage others to have an open mind and continue to research this worthy area of investigation that will potentially “redeem joy” to RDS victims all over the world.

## References

[1] Smith DE (2012). The process addictions and the new ASAM definition of addiction. J Psychoactive Drugs.

[2] Blum K, Femino J, Teitelbaum S, Giordano J, Oscar-Berman M, Gold MS (2013). Molecular neurobiology of addiction recovery: the 12 step program and fellowship.

[3] Belcher AM, Volkow ND, Moeller FG, Ferré S (2014). Personality traits and vulnerability or resilience to substance use disorders. Trends Cogn Sci.

[4] McDonald S, Darke S, Kaye S, Torok M (2013). Deficits in social perception in opioid maintenance patients, abstinent opioid users and non-opioid users. Addiction.

[5] Li M, Tian J, Zhang R, Qiu Y, Wen X, Ma X, Wang J, Xu Y, Jiang G, Huang R (2014). Abnormal cortical thickness in heroin-dependent individuals. Neuroimage.

[6] Wang X, Li B, Zhou X, Liao Y, Tang J, Liu T, Hu D, Hao W (2012). Changes in brain gray matter in abstinent heroin addicts. Drug Alcohol Depend.

[7] Luhar RB, Sawyer KS, Gravitz Z, Ruiz SM, Oscar-Berman M (2013). Brain volumes and neuropsychological performance are related to current smoking and alcoholism history. Neuropsychiatr Dis Treat.

[8] Bell RP, Foxe JJ, Ross LA, Garavan H (2013) Intact inhibitory control processes in abstinent drug abusers (I): a functional neuroimaging study in former cocaine addicts. Neuropharmacology 10.1016/j.neuropharm.2013.02.018 PMC4485393 23474013

[9] Horstmann A, Busse FP, Mathar D, Müller K, Lepsien J, Schlögl H, Kabisch S, Kratzsch J, Neumann J, Stumvoll M, Villringer A, Pleger B (2011). Obesity-related differences between women and men in brain structure and goal-directed behavior. Front Hum Neurosci.

[10] Han DH, Lyoo IK, Renshaw PF (2012). Differential regional gray matter volumes in patients with on-line game addiction and professional gamers. J Psychiatr Res.

[11] Koob GF, Volkow ND (2010). Neurocircuitry of addiction. Neuropsychopharmacology.

[12] McHugh MJ, Demers CH, Salmeron BJ, Devous MD, Stein EA, Adinoff B (2014). Cortico-amygdala coupling as a marker of early relapse risk in cocaine-addicted individuals. Front Psychiatry.

[13] Davis LM, Michaelides M, Cheskin LJ, Moran TH, Aja S, Watkins PA, Pei Z, Contoreggi C, McCullough K, Hope B, Wang GJ, Volkow ND, Thanos PK (2009). Bromocriptine administration reduces hyperphagia and adiposity and differentially affects dopamine D2 receptor and transporter binding in leptin-receptor-deficient Zucker rats and rats with diet-induced obesity. Neuroendocrinology.

[14] Rothman RB, Blough BE, Baumann MH (2008). Dual dopamine/serotonin releasers: potential treatment agents for stimulant addiction. Exp Clin Psychopharmacol.

[15] Gold MS, Blum K, Oscar-Berman M, Braverman ER (2014). Low dopamine function in attention deficit/hyperactivity disorder: should genotyping signify early diagnosis in children?. Postgrad Med.

[16] Chen TJ, Blum K, Chen AL, Bowirrat A, Downs WB, Madigan MA, Waite RL, Bailey JA, Kerner M, Yeldandi S, Majmundar N, Giordano J, Morse S, Miller D, Fornari F, Braverman ER (2011). Neurogenetics and clinical evidence for the putative activation of the brain reward circuitry by a neuroadaptagen: proposing an addiction candidate gene panel map. J Psychoactive Drugs.

[17] Blum K, Oscar-Berman M, Stuller E, Miller D, Giordano J, Morse S, McCormick L, Downs WB, Waite RL, Barh D, Neal D, Braverman ER, Lohmann R, Borsten J, Hauser M, Han D, Liu Y, Helman M, Simpatico T (2012). Neurogenetics and nutrigenomics of neuro-nutrient therapy for reward deficiency syndrome (RDS): clinical ramifications as a function of molecular neurobiological mechanisms. J Addict Res Ther.

[18] Chen TJ, Blum K, Payte JT, Schoolfield J, Hopper D, Stanford M, Braverman ER (2004). Narcotic antagonists in drug dependence: pilot study showing enhancement of compliance with SYN-10, amino-acid precursors and enkephalinase inhibition therapy. Med Hypotheses.

[19] Blum K, Chen TJ, Morse S, Giordano J, Chen AL, Thompson J, Allen C, Smolen A, Lubar J, Stice E, Downs BW, Waite RL, Madigan MA, Kerner M, Fornari F, Braverman ER (2010). Overcoming qEEG abnormalities and reward gene deficits during protracted abstinence in male psychostimulant and polydrug abusers utilizing putative dopamine D_2_ agonist therapy: part 2. Postgrad Med.

[20] Miller DK, Bowirrat A, Manka M, Miller M, Stokes S, Manka D, Allen C, Gant C, Downs BW, Smolen A, Stevens E, Yeldandi S, Blum K (2010). Acute intravenous synaptamine complex variant KB220™ “normalizes” neurological dysregulation in patients during protracted abstinence from alcohol and opiates as observed using quantitative electroencephalographic and genetic analysis for reward polymorphisms: part 1, pilot study with 2 case reports. Postgrad Med.

[21] Dani JA, De Biasi M (2013) Mesolimbic dopamine and habenulo-interpeduncular pathways in nicotine withdrawal. Cold Spring Harb Perspect Med 3(6) 10.1101/cshperspect.a012138 PMC3662347 23732854

[22] Blum K, Chen TJ, Downs BW, Bowirrat A, Waite RL, Braverman ER, Madigan M, Oscar-Berman M, DiNubile N, Stice E, Giordano J, Morse S, Gold M (2009). Neurogenetics of dopaminergic receptor supersensitivity in activation of brain reward circuitry and relapse: proposing “deprivation-amplification relapse therapy” (DART). Postgrad Med.

[23] Dahlgren A, Wargelius HL, Berglund KJ, Fahlke C, Blennow K, Zetterberg H, Oreland L, Berggren U, Balldin J (2011). Do alcohol-dependent individuals with DRD2 A1 allele have an increased risk of relapse? A pilot study. Alcohol Alcohol.

[24] Berggren U, Fahlke C, Berglund KJ, Wadell K, Zetterberg H, Blennow K, Thelle D, Balldin J (2010). Dopamine D2 receptor genotype is associated with increased mortality at a 10-year follow-up of alcohol-dependent individuals. Alcohol Alcohol.

[25] Sullivan D, Pinsonneault JK, Papp AC, Zhu H, Lemeshow S, Mash DC, Sadee W (2013). Dopamine transporter DAT and receptor DRD2 variants affect risk of lethal cocaine abuse: a gene-gene-environment interaction. Transl Psychiatry.

[26] Dackis CA, Gold MS (1985). New concepts in cocaine addiction: the dopamine depletion hypothesis. Neurosci Biobehav Rev.

[27] Blum K, Noble EP, Sheridan PJ, Montgomery A, Ritchie T, Jagadeeswaran P, Nogami H, Briggs AH, Cohn JB (1990). Allelic association of human dopamine D2 receptor gene in alcoholism. JAMA.

[28] Volkow ND, Wang GJ, Begleiter H, Porjesz B, Fowler JS, Telang F, Wong C, Ma Y, Logan J, Goldstein R, Alexoff D, Thanos PK (2006). High levels of dopamine D2 receptors in unaffected members of alcoholic families: possible protective factors. Arch Gen Psychiatry.

[29] Blum K, Braverman ER, Wood RC, Gill J, Li C, Chen TJ, Taub M, Montgomery AR, Sheridan PJ, Cull JG (1996). Increased prevalence of the Taq I A1 allele of the dopamine receptor gene (DRD2) in obesity with comorbid substance use disorder: a preliminary report. Pharmacogenetics.

[30] Wang GJ, Volkow ND, Logan J, Pappas NR, Wong CT, Zhu W, Netusil N, Fowler JS (2001). Brain dopamine and obesity. Lancet.

[31] Avena NM, Rada P, Hoebel BG (2008). Evidence for sugar addiction: behavioral and neurochemical effects of intermittent, excessive sugar intake. Neurosci Biobehav Rev.

[32] Bocarsly ME, Hoebel BG, Paredes D, von Loga I, Murray SM, Wang M, Arolfo MP, Yao L, Diamond I, Avena NM (2014). GS 455534 selectively suppresses binge eating of palatable food and attenuates dopamine release in the accumbens of sugar-bingeing rats. Behav Pharmacol.

[33] Gardner EL (2011). Addiction and brain reward and anti-reward pathways. Adv Psychosom Med.

[34] Koob GF (2009). Neurobiological substrates for the dark side of compulsivity in addiction. Neuropharmacology.

[35] Elman I, Borsook D, Volkow ND (2013). Pain and suicidality: insights from reward and addiction neuroscience. Prog Neurobiol.

[36] Blum K, Chen AL, Giordano J, Borsten J, Chen TJ, Hauser M, Simpatico T, Femino J, Braverman ER, Barh D (2012). The addictive brain: all roads lead to dopamine. J Psychoactive Drugs.

[37] Previc FH (2009). The dopaminergic mind in human evolution and history.

[38] Horwitz B, Swedo SE, Grady CL, Pietrini P, Schapiro MB, Rapoport JL, Rapoport SI (1991). Cerebral metabolic pattern in obsessive-compulsive disorder: altered intercorrelations between regional rates of glucose utilization. Psychiatry Res.

[39] Raghanti MA, Stimpson CD, Marcinkiewicz JL, Erwin JM, Hof PR, Sherwood CC (2008). Cortical dopaminergic innervation among humans, chimpanzees, and macaque monkeys: a comparative study. Neuroscience.

[40] Adams DK, Sewell MA, Angerer RC, Angerer LM (2011). Rapid adaptation to food availability by a dopamine-mediated morphogenetic response. Nat Commun.

[41] Boehmler W, Obrecht-Pflumio S, Canfield V, Thisse C, Thisse B, Levenson R (2004). Evolution and expression of D2 and D3 dopamine receptor genes in zebrafish. Dev Dyn.

[42] Noble EP, Blum K, Ritchie T, Montgomery A, Sheridan PJ (1991). Allelic association of the D2 dopamine receptor gene with receptor-binding characteristics in alcoholism. Arch Gen Psychiatry.

[43] Blum K, Gardner E, Oscar-Berman M, Gold M (2012). “Liking” and “wanting” linked to reward deficiency syndrome (RDS): hypothesizing differential responsivity in brain reward circuitry. Curr Pharm Des.

[44] Blum K, Wood RC, Braverman ER, Chen TJ, Sheridan PJ (1995). The D2 dopamine receptor gene as a predictor of compulsive disease: Bayes’ theorem. Funct Neurol.

[45] Sussman S, Stacy AW, Johnson CA, Pentz MA, Robertson E (2004). A transdisciplinary focus on drug abuse prevention: an introduction. Subst Use Misuse.

[46] Peña CJ, Bagot RC, Labonté B, Nestler EJ (2014) Epigenetic signaling in psychiatric disorders. J Mol Biol 10.1016/j.jmb.2014.03.016 PMC4177298 24709417

[47] Gueorguieva R, Wu R, Donovan D, Rounsaville BJ, Couper D, Krystal JH, O’Malley SS (2011). Baseline trajectories of drinking moderate acamprosate and naltrexone effects in the COMBINE study. Alcohol Clin Exp Res.

[48] Cano-Cebrián MJ, Zornoza-Sabina T, Guerri C, Polache A, Granero L (2003). Local acamprosate modulates dopamine release in the rat nucleus accumbens through NMDA receptors: an in vivo microdialysis study. Naunyn Schmiedebergs Arch Pharmacol.

[49] Shelton KL, Hendrick ES, Beardsley PM (2013). Efficacy of buspirone for attenuating cocaine and methamphetamine reinstatement in rats. Drug Alcohol Depend.

[50] Blum K, Oscar-Berman M, Dinubile N, Giordano J, Braverman ER, Truesdell CE, Barh D, Badgaiyan R (2013). Coupling genetic addiction risk score (GARS) with electrotherapy: fighting iatrogenic opioid dependence. J Addict Res Ther.

[51] Bogomolova EV, Rauschenbach IY, Adonyeva NV, Alekseev AA, Faddeeva NV, Gruntenko NE (2010). Dopamine down-regulates activity of alkaline phosphatase in drosophila: the role of D2-like receptors. J Insect Physiol.

[52] Maguire EP, Macpherson T, Swinny JD, Dixon CI, Herd MB, Belelli D, Stephens DN, King SL, Lambert JJ (2014). Tonic inhibition of accumbal spiny neurons by extrasynaptic α4βδ GABAA receptors modulates the actions of psychostimulants. J Neurosci.

[53] Czoty PW, Nader MA (2013). Effects of dopamine D2/D3 receptor ligands on food-cocaine choice in socially housed male cynomolgus monkeys. J Pharmacol Exp Ther.

[54] Radke AK, Gewirtz JC (2012). Increased dopamine receptor activity in the nucleus accumbens shell ameliorates anxiety during drug withdrawal. Neuropsychopharmacology.

[55] Thanos PK, Michaelides M, Umegaki H, Volkow ND (2008). D2R DNA transfer into the nucleus accumbens attenuates cocaine self-administration in rats. Synapse.

[56] Thanos PK, Rivera SN, Weaver K, Grandy DK, Rubinstein M, Umegaki H, Wang GJ, Hitzemann R, Volkow ND (2005). Dopamine D2R DNA transfer in dopamine D2 receptor-deficient mice: effects on ethanol drinking. Life Sci.

[57] Thanos PK, Volkow ND, Freimuth P, Umegaki H, Ikari H, Roth G, Ingram DK, Hitzemann R (2001). Overexpression of dopamine D2 receptors reduces alcohol self-administration. J Neurochem.

[58] Crunelle CL, Schulz S, de Bruin K, Miller ML, van den Brink W, Booij J (2009). Dose-dependent and sustained effects of varenicline on dopamine D2/3 receptor availability in rats. Addict Biol.

[59] van Rijn RM, Brissett DI, Whistler JL (2010). Dual efficacy of delta opioid receptor-selective ligands for ethanol drinking and anxiety. J Pharmacol Exp Ther.

[60] Ribeiro SJ, Ciscato JG, de Oliveira R, de Oliveira RC, D’Angelo-Dias R, Carvalho AD, Felippotti TT, Rebouças EC, Castellan-Baldan L, Hoffmann A, Corrêa SA, Moreira JE, Coimbra NC (2005). Functional and ultrastructural neuroanatomy of interactive intratectal/tectonigral mesencephalic opioid inhibitory links and nigrotectal GABAergic pathways: involvement of GABAA and mu1-opioid receptors in the modulation of panic-like reactions elicited by electrical stimulation of the dorsal midbrain. J Chem Neuroanat.

[61] Carvalho RC, Fukushiro DF, Helfer DC, Callegaro-Filho D, Trombin TF, Zanlorenci LH, Sanday L, Silva RH, Frussa-Filho R (2011). Long-term haloperidol treatment (but not risperidone) enhances addiction-related behaviors in mice: role of dopamine D2 receptors. Eur Neuropsychopharmacol.

[62] Graham DL, Hoppenot R, Hendryx A, Self DW (2007). Differential ability of D1 and D2 dopamine receptor agonists to induce and modulate expression and reinstatement of cocaine place preference in rats. Psychopharmacology (Berl).

[63] Milivojevic N, Krisch I, Sket D, Zivin M (2004). The dopamine D1 receptor agonist and D2 receptor antagonist LEK-8829 attenuates reinstatement of cocaine-seeking in rats. Naunyn Schmiedebergs Arch Pharmacol.

[64] Caine SB, Negus SS, Mello NK, Patel S, Bristow L, Kulagowski J, Vallone D, Saiardi A, Borrelli E (2002). Role of dopamine D2-like receptors in cocaine self-administration: studies with D2 receptor mutant mice and novel D2 receptor antagonists. J Neurosci.

[65] Schmidt LG, Kuhn S, Smolka M, Schmidt K, Rommelspacher H (2002). Lisuride, a dopamine D2 receptor agonist, and anticraving drug expectancy as modifiers of relapse in alcohol dependence. Prog Neuropsychopharmacol Biol Psychiatry.

[66] Weissenborn R, Deroche V, Koob GF, Weiss F (1996). Effects of dopamine agonists and antagonists on cocaine-induced operant responding for a cocaine-associated stimulus. Psychopharmacology (Berl).

[67] Lawford BR, Young RM, Rowell JA, Qualichefski J, Fletcher BH, Syndulko K, Ritchie T, Noble EP (1995). Bromocriptine in the treatment of alcoholics with the D2 dopamine receptor A1 allele. Nat Med.

[68] Ng GY, George SR (1994). Dopamine receptor agonist reduces ethanol self-administration in the ethanol-preferring C57BL/6J inbred mouse. Eur J Pharmacol.

[69] Blum K, Elston SF, DeLallo L, Briggs AH, Wallace JE (1983). Ethanol acceptance as a function of genotype amounts of brain [Met] enkephalin. Proc Natl Acad Sci U S A.

[70] Ungless MA (2004). Dopamine: the salient issue. Trends Neurosci.

[71] Suto N, Ecke LE, Wise RA (2009). Control of within-binge cocaine-seeking by dopamine and glutamate in the core of nucleus accumbens. Psychopharmacology (Berl).

[72] Suto N, Wise RA (2011). Satiating effects of cocaine are controlled by dopamine actions in the nucleus accumbens core. J Neurosci.

[73] Willuhn I, Burgeno LM, Groblewski PA, Phillips PE (2014). Excessive cocaine use results from decreased phasic dopamine signaling in the striatum. Nat Neurosci.

[74] Caprioli D, Calu D, Shaham Y (2014). Loss of phasic dopamine: a new addiction marker?. Nat Neurosci.

[75] Grabowski J, Rhoades H, Stotts A, Cowan K, Kopecky C, Dougherty A, Moeller FG, Hassan S, Schmitz J (2004). Agonist-like or antagonist-like treatment for cocaine dependence with methadone for heroin dependence: two double-blind randomized clinical trials. Neuropsychopharmacology.

[76] Dackis CA, Gold MS (1985). Bromocriptine as treatment of cocaine abuse. Lancet.

[77] Renthal W, Nestler EJ (2008). Epigenetic mechanisms in drug addiction. Trends Mol Med.

[78] Peña CJ, Bagot RC, Labonté B, Nestler EJ (2014). Epigenetic signaling in psychiatric disorders. J Mol Biol.

[79] Szyf M (2013). DNA methylation, behavior and early life adversity. J Genet Genomics.

[80] Kofink D, Boks MP, Timmers HT, Kas MJ (2013). Epigenetic dynamics in psychiatric disorders: environmental programming of neurodevelopmental processes. Neurosci Biobehav Rev.

[81] Gonzalez-Pardo H, Perez AM (2013). Epigenetics and its implications for psychology. Psicothema.

[82] Borsten J (2013). Dopamine for dinner: an easy-to-follow 4 week meal plan to help break the cycle of addiction.

